# First identification of mammalian orthoreovirus type 3 by gut virome analysis in diarrheic child in Brazil

**DOI:** 10.1038/s41598-019-55216-5

**Published:** 2019-12-09

**Authors:** Ulisses Alves Rosa, Geovani de Oliveira Ribeiro, Fabiola Villanova, Adriana Luchs, Flávio Augusto de Pádua Milagres, Shirley Vasconcelos Komninakis, Roozbeh Tahmasebi, Márcia Cristina Alves Brito Sayão Lobato, Rafael Brustulin, Rogério Togisaki das Chagas, Maria de Fátima Neves dos Santos Abrão, Cassia Vitória de Deus Alves Soares, Rory J Tinker, Ramendra Pati Pandey, V. Samuel Raj, Ester Cerdeira Sabino, Xutao Deng, Eric Delwart, Antonio Charlys da Costa, Élcio Leal

**Affiliations:** 1 Institute of Biological Sciences, Federal University of Para, Para, 66075-000 Brazil; 2 Enteric Disease Laboratory, Virology Center, Adolfo Lutz Institute, Sao Paulo, 01246-000 Brazil; 3 Secretary of Health of Tocantins, Tocantins, 77453-000 Brazil; 4 Institute of Biological Sciences, Federal University of Tocantins, Tocantins, 77001-090 Brazil; 5 Public Health Laboratory of Tocantins State (LACEN/TO), Tocantins, 77016-330 Brazil; 6 Postgraduate Program in Health Science, Faculty of Medicine of ABC, Santo André, 09060-870 Brazil; 7 Retrovirology Laboratory, Federal University of São Paulo, São Paulo, 04023-062 Brazil; 8 Instituto de Medicina Tropical, Universidade de São Paulo, São Paulo, 05403-000 Brazil; 9 Polytechnic School of University of Sao Paulo, Sao Paulo, Brazil; 10 Faculty of Biology, Medicine and Health, University of Manchester, Manchester, M13 9PL UK; 11 Centre for Drug Design Discovery and Development (C4D), SRM University, Delhi-NCR, Rajiv Gandhi Education City, Sonepat, 131 029 Haryana India; 12 LIM/46, Faculdade de Medicina, Universidade de São Paulo, São Paulo, 01246-903 Brazil; 13 Vitalant Research Institute, San Francisco, CA 94143 USA; 14 Department Laboratory Medicine, University of California San Francisco, San Francisco, CA 94143 USA

**Keywords:** Viral infection, Phylogenomics, Molecular medicine

## Abstract

Diarrhea remains one of the most common causes of deaths in children. Although many studies have investigated the prevalence of enteric pathogens around the globe some diarrheal episodes remain unexplained. It is possible that some yet-unidentified viral agents could be related to these cases of gastroenteritis. By using viral metagenomics techniques, we screened 251 fecal samples of children between 0.5 to 2.5-year-old with acute diarrhea not associated with common pathogens. These children live in rural areas and have different levels of contact with animals such as pigs, cows and bats. Here we report a complete genome of one mammalian orthoreovirus (MRV) type 3, denoted TO-151/BR, detected in a female child in the state of Tocantins (north of Brazil). Brazilian TO-151/BR strain was classified as MRV-3 based on S1 phylogeny and was closely related to porcine Asian strains. Phylogenetic analyses showed that other segments were more similar to MRV-3s of different geographic locations and hosts, including human and bats, highlighting genome reassortment and lack of host-specific barriers. This is the first report of MRV-3 in South America and a hypothesis of a silent long-term circulation of this virus in Brazil has been raised.

## Introduction

MRV is non-enveloped virus comprises of genome size of approximately 23,500 base pairs contains 10 segmented dsRNA genome of three different sizes: three large segments (L1, L2 and L3), medium segments (M1, M2 and M3), and small segments (S1, S2, S3 and S4). Orthoreoviruses belong to genus *Orthoreovirus* of Reoviridae family. First incidence of Mammalian orthoreoviruses (MRVs) infection was reported in 1950^1^. Respiratory and gastrointestinal tract are common route for MRVs infection in vertebrates. Based on the ability to induce cell–cell fusion and syncytium formation^2,3^ orthoreoviruses can be further divided into two subgroups, fusogenic and non-fusogenic. Members of MRV further classified into four serotypes (type 1 Lang, type 2 Jones, type 3 Dearing, and type 4 Ndelle) and are nonfusogenic^2–4^.

MRVs are associated to gastrointestinal and respiratory illness and have been isolated from a wide variety of mammalian species including bats, minks, pigs and humans^5,6^. In humans, MRV infections usually cause mild respiratory and enteric infections, displaying high fever, diarrhea and vomiting symptoms^7^. However, recently, reports associating MRV to more severe illnesses, including pneumonia, encephalitis and respiratory distress syndrome, have been increasing^8,9^.

MRV has been detected in humans worldwide, including Malaysia, Indonesia, Hong Kong, USA, Canada and France^8–12^; nevertheless, not yet identified in Latin America continent. Type 3 MRV (MRV-3) was isolated and characterized in the USA having caused meningitis in children^12^ and diarrhea^4^. Additionally, MRV-3 has been recently isolated from piglets with severe diarrhea and respiratory symptoms in Asia, USA and Europe^5,6,13^. In the present study, we report the detection of MRV-3 and whole genomic characterization in Brazil for the first time. Near full genome of MRV-3 was isolated from a child with vomiting and diarrhea, and an evolutionary analysis was conducted. The present investigation has the potential to innovate in the orthoreovirus genomes knowledge and understanding, highlighting its zoonotic potential.

## Materials and Methods

### Patients

This study was part of surveillance program carried out in the state of Tocantins, north Brazil, from 2010 to 2016 and details regarding the selection of patients were described previously^14–18^. Briefly, a total of 251 fecal specimens were collected between 2010 and 2016 and screened for enteric pathogens (i.e., rotavirus and norovirus), bacteria (i.e., *Escherichia coli* and *Salmonella* sp.), endoparasites (i.e., Giardia sp.), and helminthes, using conventional culture techniques and commercial enzyme immunoassays. Patients ranging from 0.5 to 2.5 years old, with the exception one 50-year-old individual, suffered from acute gastroenteritis at the time of sampling. To identify possible undetected enteric viruses, NGS techniques were applied to all 251 samples using the method described below. Rotaviruses (n = 112), adenoviruses (n = 84), norovirus (n = 39), classical astroviruses (n = 8), and sapovirus (n = 8) were identified in some of these subjects. The patient infected by MRV-3 was born on July 23, 2013, and was an inhabitant of the rural area of Araguaina, a municipality located in the Brazilian state of Tocantins. The patient was diagnosed with gastroenteritis and submitted to screening the main enteric pathogens (rotavirus, *Escherichia coli, Salmonella* spp., *Giardia* sp. and helminths) using traditional detection methods (i.e., culture techniques and commercial enzyme immunoassay). No common enteric pathogens were detected. Therefore, to identify the pathogen-associated with symptomology, NGS techniques were applied.

### Ethical approval

All procedures performed in studies involving human participants were in accordance with the ethical standards of the institutional and/or national research committee and with the 1964 Helsinki Declaration and its later amendments or comparable ethical standards. Informed consent was obtained from the adult individual and from all parents or guardians of children participants involved in the study. Ethics Committee approval was granted by Faculdade de Medicina da Universidade de São Paulo (CAAE: 53153916.7.0000.0065), and Centro Universitário Luterano de Palmas — ULBRA (CAAE: 53153916.7.3007.5516).

### Sample processing

The protocol used to perform deep sequencing was a combination of several protocols normally applied to viral metagenomics and/or virus discovery previously as^19^. Briefly, 50 mg of the human fecal sample was diluted in 500 μL of Hank’s buffered salt solution (HBSS) and added to a 2 mL impact-resistant tube containing lysing matrix C (MP Biomedicals, Santa Ana, CA, USA), and homogenized in a FastPrep-24 5 G Homogenizer (MP biomedicals, USA). The homogenized sample was centrifuged at 12,000 × *g* for 10 min and approximately 300 μL of the supernatant was then percolated through a 0.45 μm filter (Merck Millipore, Billerica, MA, USA) to remove eukaryotic- and bacterial-cell-sized particles. Approximately 100 μL, roughly equivalent to one-fourth of the volume of the tube, of cold PEG-it Virus Precipitation Solution (System Biosciences, Palo Alto, CA, USA) was added to the filtrate and the contents of the tube were gently mixed and then incubated at 4 °C for 24 h. After the incubation period, the mixture was centrifuged at 10,000 × *g* for 30 min at 4 °C. Following centrifugation, the supernatant (~350 μL) was discarded. The pellet, rich in viral particles was treated with a combination of nuclease enzymes (TURBO DNase and RNase Cocktail Enzyme Mix-Thermo Fischer Scientific, Waltham, MA, USA; Baseline-ZERO DNase-Epicentre, Madison, WI, USA; Benzonase-Darmstadt, Darmstadt, Germany; and RQ1 RNase-Free DNase and RNase A Solution-Promega, Madison, WI, USA) to digest the unprotected nucleic acids. The resulting mixture was subsequently incubated at 37 °C for 2 h. After incubation, viral nucleic acids were extracted using a ZR & ZR-96 Viral DNA/RNA Kit (Zymo Research, Irvine, CA, USA) according to the manufacturer’s instructions. The cDNA synthesis was performed with an AMV reverse transcription reagent (Promega, Madison, WI, USA). A second strand cDNA synthesis was performed using a DNA Polymerase I Large (Klenow) Fragment (Promega). Subsequently, a Nextera XT Sample Preparation Kit (Illumina, San Diego, CA, USA) was used to construct a DNA library, which was identified using dual barcodes. For the size range, Pippin Prep (Sage Science, Inc., Beverly, MA, USA) was used to select a 300 bp insert (range 200–400 bp). The library was deep-sequenced using a Hi-Seq. 2500 Sequencer (Illumina, CA, USA) with 126 bp ends. Bioinformatics analysis was performed according to the protocol previously described by Deng *et al*.^20^. The contigs, including sequences of rotaviruses, as well as enteric viruses, human, fungi, bacteria, and others, sharing a percent nucleotide identity of 95% or less were assembled from the obtained sequence reads by de novo assembly. The resulting singlets and contigs were analyzed using BLASTx to search for similarity to viral proteins in GenBank. The contigs were compared to the GenBank non-redundant nucleotide and protein databases (BLASTn and BLASTx). After identification of the viruses, a reference template sequence was used for mapping the full-length genome with Geneious R9 software (Biomatters Ltd L2, 18 Shortland Street Auckland, 1010, New Zealand). Based on the best hits of the BLASTx searches, MRV genomes were chosen for further analyses.

### Alignment and phylogenetic analysis

These genomes were aligned using Clustal X software^21^. Subsequently, a phylogenetic tree was constructed using the maximum likelihood approach and branch support values were assessed using the Shimodaira–Hasegawa test. All trees were inferred using FastTree software^22^ and the GTR model and gamma distribution were selected according to the likelihood ratio test (LRT) implemented in the jModeltest software^23^. Analyses were performed using 1000 replications. The similarity analysis was conducted in Similarity Matrix tool of MegaX software^24^.

Nucleotide sequences determined in this study have been deposited in GenBank under the accession numbers MN022930-MN022939.

## Results and Discussion

**Figure 1:**
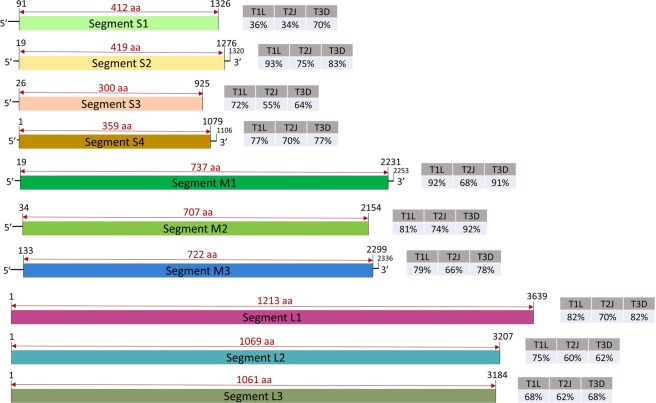
Segmented genome of the MRV-3 strain TO-151/BR. Colored rectangles indicate coding region of each genomic segment of MRV-3. Numbers flanking each segment indicate the start and ending of the coding region. Numbers above the double headed arrows represent size in amino acids (aa) of the coding region. Dark lines indicate the size of 5′ and 3′ terminal repeats. Gray boxes on the right side of each segment indicate the, the nucleotide identity of TO-151/BR with the strains MRV-1 Lang (T1L), MRV-2 Jones (T2J) and MRV-3 Dearing (T3D).

The nearly-complete segmented genome the novel MRV-3 strain TO-151/BR is shown in Fig. 1. A more in-depth molecular characterization was performed, highlighting some features in comparison to other strains. Pairwise nucleotide identity comparison was conducted of all ten segments between strain TO-151/BR and the three prototype strains (MRV-1 Lang (T1L); MRV-2 Jones (T2J); and MRV-3 Dearing (T3D). The TO-151/BR strain is more similar to T1L strain in segments S2, S3, M1, M3 and L2, by other hand, in segments S1 and M2 is more identical to T3D strain (Fig. 1). Additionally, TO-151/BR strain has same nucleotide identity percent with T1L and T3D strains in segments S4, L1 and L3.

MRV are divided into three genotypes (MRV-1, MRV-2 and MRV-3) and genotyping are determined by segment S1^25^. Based on S1 phylogeny, Brazilian TO-151/BR MRV-3 strain belonged to the lineage b of the MRV3 and was closely related to Asian porcine MRV-3s strains associated with diarrhea in pig. It shares the highest nucleotide (95.7%) identity with the porcine MRV-3 SC-A strain (DQ911244) detected from a pig with diarrhea symptoms in China (Table 1). The other segments L1, L3, M1, M3, S2, S3 and S4 of the Brazilian TO-151/BR MRV-3 strain were related to a wild range of species, including pig, human, masked civet cat, murine and bat strains (Table 1).

**Table 1:** Highest nucleotide identities for each gene segment of the Brazilian TO-151/BR MRV-3 strain.

Segment	Strain	Similarity	Host	Country	Genbank ID	Year
M1	GD-1	91.6%	Pig	China	JX486060	2012
M2	Abney	94.5%	Human	USA	GU589581	1957
M3	MPC/04	91.8%	Masked Civet cat	China	GQ468271	2004
L1	SC-A	93.9%	Pig	China	DQ997719	2006
L2	T3	94.2%	Mouse	France	AF378007	1961
L3	SC-A	97.4%	Pig	China	EF029088	2006
S1	SC-A	95.7%	Pig	China	DQ911244	2006
S2	WIV7	97.8%	Bat	China	KT444559	2011
S3	FS-03	93.5%	Pig	USA	KM820762	2014
S4	MPC/04	91.9%	Masked Civet cat	China	GQ468275	2004

**Figure 2:**
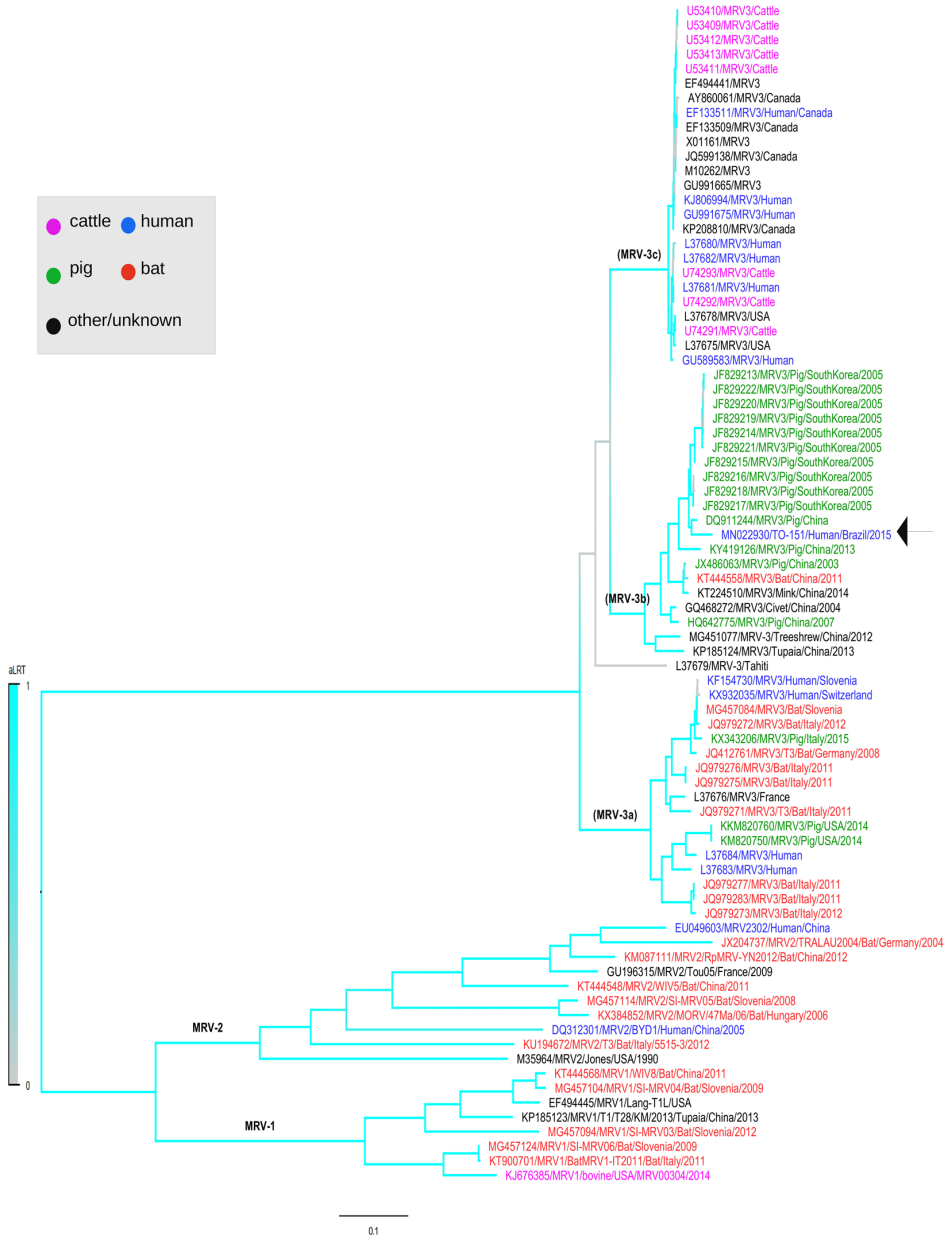
Phylogenetic tree of the complete S1 segment of the Brazilian TO-151/BR MRV-3 strain (indicated in a arrow). The tree was performed using the maximum likelihood method GTR-G model within the jModeltest software with a bootstrap of 1000 replicates. Accession number, species, isolate, country and year are indicated for each strain. Different hosts are highlighted in different colors. Lower case a,b and c represent three different MRV-3 lineages.

Since the above analysis of nucleotide identity not provide the evolutionary relatedness, we also performed a detailed phylogenetic analysis using maximum likelihood approach (Fig. 2, Supplementary Figs. S1–S9). The phylogenetic analysis confirmed the observed discrepancies in the clustering of these genomic genes of MRV-3. This data reflects a pattern of topological incongruence caused by reassortment^26^. A study conducted whit MRV-1 strains obtained from bats in Italy also reported this observation^5^. High divergence was observed between the Brazilian TO-151/BR strain and other MRV-3 isolates available in GenBank in the phylogenetic trees, suggesting that this virus may be circulating in Brazil for a long time. Nevertheless, the lack of Brazilian MRV-3 sequences limited to draw any robust conclusions. The present study also highlights the capacity of MRVs to remain in silent circulation in human and animal populations^10,27^. Surveillance of MRV among distinct mammalian species can considerably increase the power of emergent zoonotic diseases strategies.

The data obtained here suggests that TO-151/BR strain may have arisen through multiple reassortment events of MRV-3s from varied origins. Due to the segmented genome of the MRV, different combinations of the segments could be obtained from reassortment and result in the formation of novel genotypes. Several studies have been reporting MRVs reassortments^5,7,13,25,26^. Other members of *Reoviridae* family such as rotaviruses also share this particular evolutionary pathway^28^. The capacity of important human and animal pathogens such as influenza A viruses and rotavirus A strains to reassert, has important implications for their ongoing evolution and impact on global health It is likely that MRVs also display this potential^2,8,10,26,27,29^.

Due to lack of host specificity, MRVs infection poses a major concern for zoonotic transmission as we all spread of infection in human and animal hosts^27,30^. In current study we have conducted a phylogenetic analysis. Our studies establish a close relationship between Brazilian TO-151/BR MRV-3 strain and strains isolated from different species, primarily from pigs, underscore a high probability of zoonotic transmission. Based on current study, we predict that a genome reassortment may have occurred in the animal host(s) prior to transmission in the human host. However, at this time we could not ascertain the exact direction of transmission events and could not determine unequivocally the origin of the MRV-3 strain found in humans. Further studies are warranted to address these two questions with confidence. Notably, MRV-3 strain reported and analyzed in current study, was detected in a child staying in rural area. In rural population, propensity of interspecies transmission is fairly high^27,30^. High propensity of interspecies transmission in rural population is due to poor hygiene of inhabitants, close proximity to animals and common source of drinking water. Children have close interactions with pets with limited hygiene habits. Therefore, children are more prone to infection compared to adult^6,9,17,18,31^.

Contradicting reports fail to establish the pathogen role of MRV-3 unequivocally^7,8,10,12,25,31–33^. MRV-3 infection may be asymptomatic or symptomatic^7,8,10,12,31,33^. Further, complexity is compounded by MRV-3 infection in association with other pathogens in human and animals. Pertaining to our study, gastroenteritis symptoms was observed in patient TO-151/BR, and may be associated to one (or more) of the enteric viruses (i.e., Rotavirus A, Adenovirus and Norovirus), which was detected in the fecal sample (see the number of reads in the figure S10)^14,15,17,18^. Alternatively, presence of MRV-3 in feces may be due to dietary consumption of infected porcine meat^34^. Although virome analysis is powerful tool^32^, it could not performed accurately, due to lack of medical records of the patients or epidemiological background information on potential contact with animals or consumption of contaminated food. Present study also fails to establish the epidemiological connection between the patient and the virus.

In summary, present study adopted a NGS analyses approach to identify MRV-3 infection in human specimens, obtained from South America. Current study contributes to growing database of the geographic distribution and molecular diversity of MRV. Generation of additional sequence data in combination with functional studies are warranted to understand the ecology, epidemiology, and evolution of MRV.

## Supplementary Information

supplementary information
