# Supplementary material for: First identification of mammalian orthoreovirus type 3 by gut virome analysis in diarrheic child in Brazil
Source: Sci Rep. 2019 Dec 9;9:18599. doi: 10.1038/s41598-019-55216-5 (PMC6901473; doi:10.1038/s41598-019-55216-5)
Supplement: Supplementary file 1 — supplementary information [file 41598_2019_55216_MOESM1_ESM.pdf]

Supplementary material;

First identification of mammalian orthoreovirus type 3 by gut virome analysis in diarrheic child in Brazil

Ulisses Alves Rosa[1], Geovani de Oliveira Ribeiro[1], Fabiola Villanova[1], Adriana Luchs[2], Flávio Augusto de Pádua Milagres[3,4,5], Shirley Vasconcelos Komninakis[6,7], Roozbeh Tahmasebi[8,9], Márcia Cristina Alves Brito Sayão Lobato[3,5], Rafael Brustulin[3,4,5], Rogério Togisaki das Chagas[3,5], Maria de Fátima Neves dos Santos Abrão[3,5], Cassia Vitória de Deus Alves Soares[3,5], Rory J Tinker[8,10], Ramendra Pati Pandey[11], V. Samuel Raj[11], Ester Cerdeira Sabino[8,12], Xutao Deng[13,14], Eric Delwart[13,14], Antonio Charlys da Costa[8,*,‡] and Élcio Leal[1,*,‡]

[1]Institute of Biological Sciences, Federal University of Para, Para 66075-000, Brazil; ualvesfisio@yahoo.com.br (U.A.R.); fevface@gmail.com (F.V.); geovanibiotec@gmail.com (G.O.R.); elcioleal@gmail.com (É.L.)

[2]Enteric Disease Laboratory, Virology Center, Adolfo Lutz Institute, Sao Paulo 01246-000, Brazil; driluchs@gmail.com

[3]Secretary of Health of Tocantins, Tocantins 77453-000, Brazil; flaviomilagres@uft.edu.br (F.A.d.P.M); eumarciaalvesbrito@gmail.com (M.C.A.B.S.L.); eu3rafael@gmail.com (R.B.); chagastogisaki@hotmail.com (R.T.d.C.); fatima_abrao@yahoo.com.br (M.d.F.N.d.S.A.); cassiavitoriaalves@gmail.com (C.V.d.D.A.S.)

[4]Institute of Biological Sciences, Federal University of Tocantins, Tocantins 77001-090, Brazil

[5]Public Health Laboratory of Tocantins State (LACEN/TO), Tocantins 77016-330, Brazil.

[6]Postgraduate Program in Health Science, Faculty of Medicine of ABC, Santo André 09060-870, Brazil; skomninakis@yahoo.com.br (S.V.K.)

[7]Retrovirology Laboratory, Federal University of São Paulo, São Paulo 04023-062, Brazil

[8]Instituto de Medicina Tropical, Universidade de São Paulo, São Paulo 05403-000, Brazil; charlysbr@yahoo.com.br (A.C.d.C); sabinoec@gmail.com (E.C.S.); rorytinker2011@gmail.com (R.J.T)

[9]Polytechnic School of University of Sao Paulo, Sao Paulo, Brazil; roozbeh@usp.br (R.T.)

[10]Faculty of Biology, Medicine and Health, University of Manchester, Manchester, M13 9PL, UK

[11]Centre for Drug Design Discovery and Development (C4D), SRM University, Delhi-NCR, Rajiv Gandhi Education City, Sonepat - 131 029, Haryana, India; ramendra.pandey@gmail.com (R.P.P.), directorcd4@srmuniversity.ac.in (V.S.R.)

[12]LIM/46, Faculdade de Medicina, Universidade de São Paulo, São Paulo 01246-903, Brazil,

[13]Vitalant Research Institute, San Francisco, CA 94143, USA; xdeng@vitalant.org (X.D.); eric.delwart@ucsf.edu (E.D)

[14]Department Laboratory Medicine, University of California San Francisco, San Francisco, CA 94143, USA

Keywords: **Virus identification, Mammalian orthoreovirus, Virome, Pediatric, Gastroenteritis, Next generation sequencing.**

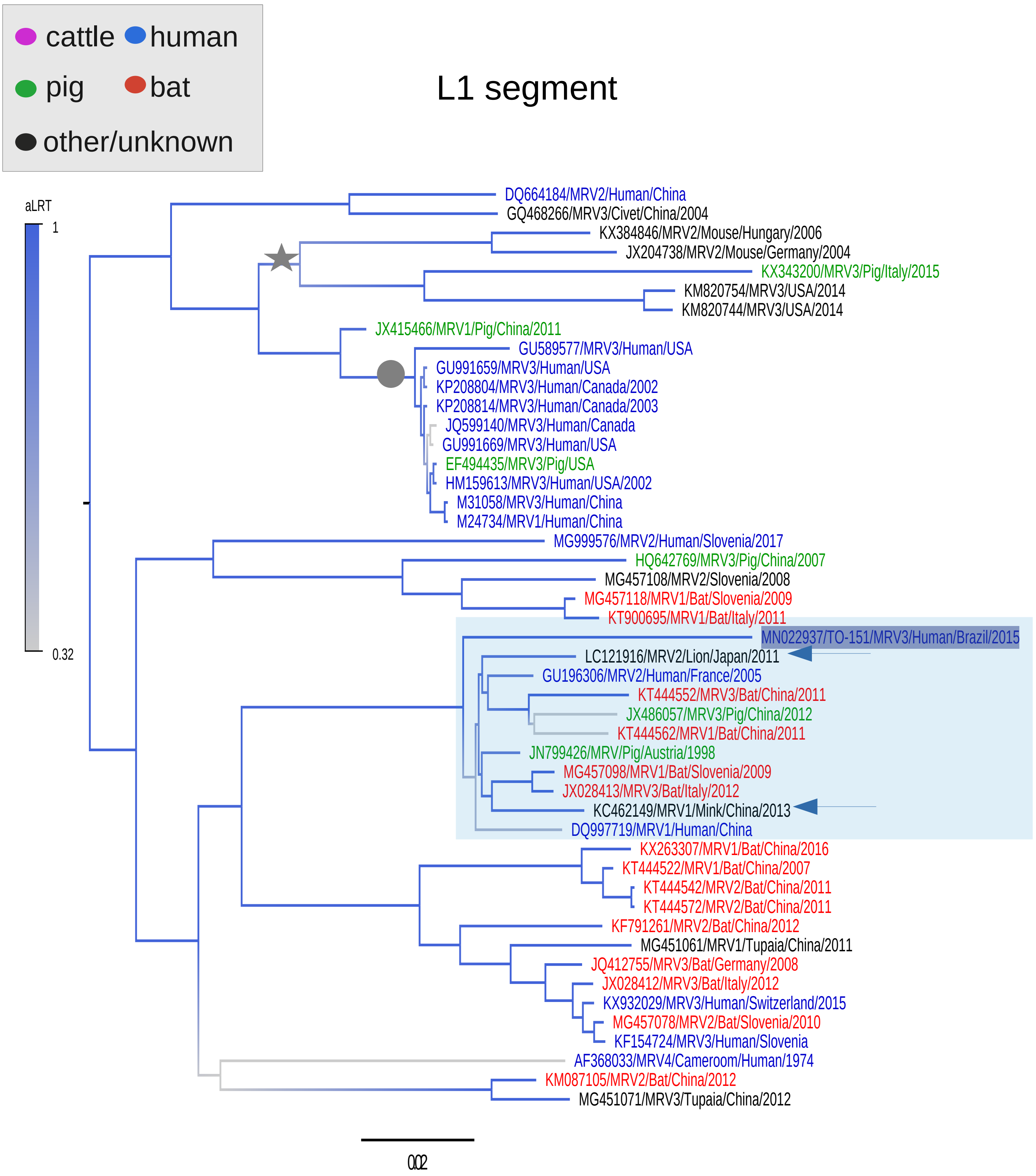

Figure S1. Phylogenetic tree inferred using the L1 segment of MRV3. Phylogenetic tree of the complete L1 segment (3639 bp) of the Brazilian TO-151/BR MRV3 strain (highlighted in the tree). The tree was performed using the maximum likelihood method GTR-G model within the jModeltest software with a bootstrap of 1000 replicates. Accession number, species, isolate, country and year are indicated for each strain. Different hosts are highlighted in different colors.

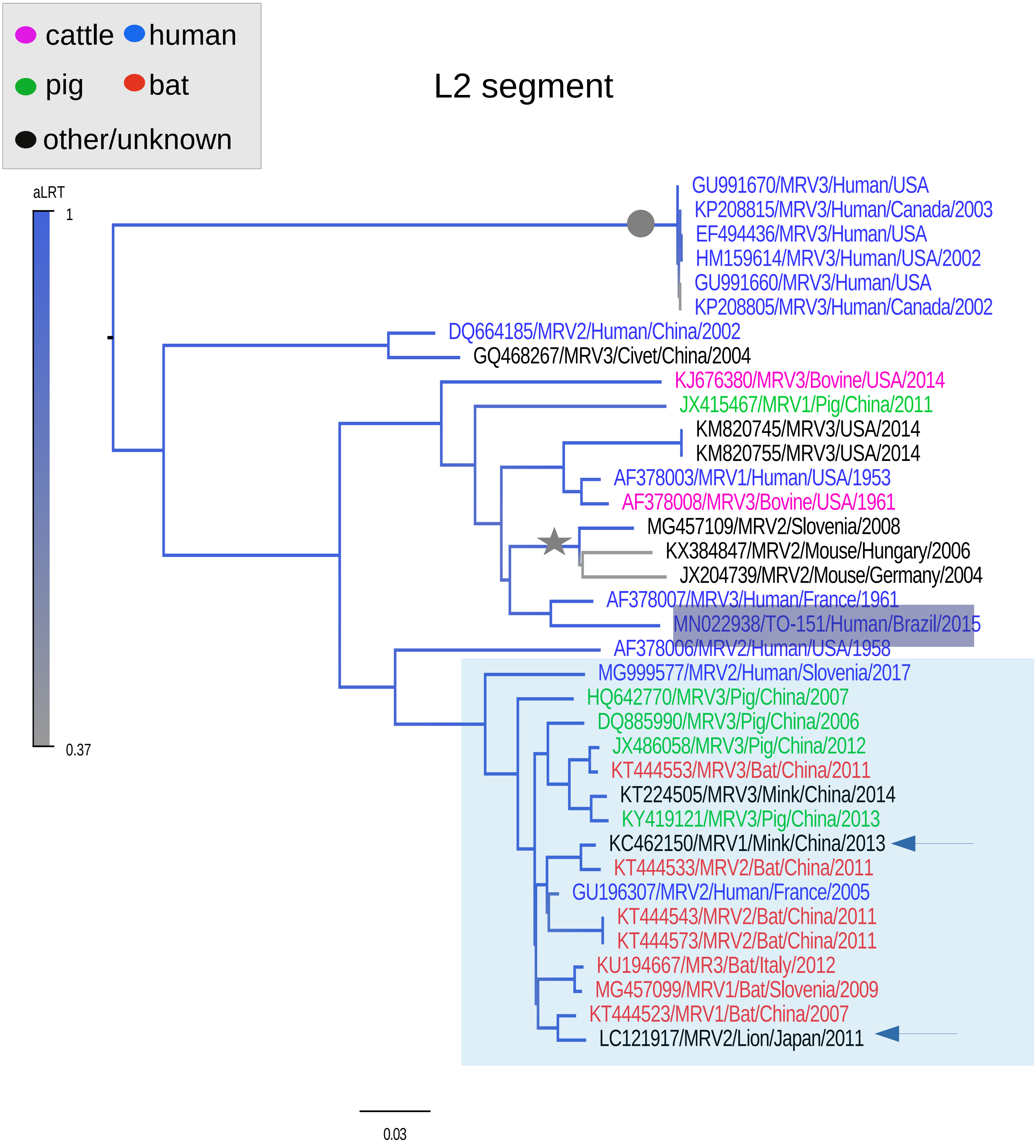

Figure S2. Phylogenetic tree inferred using the L2 segment of MRV3. Phylogenetic tree of the complete L2 segment (3207 bp) of the Brazilian TO-151/BR MRV3 strain (highlighted in the tree). The tree was performed using the maximum likelihood method GTR-G model within the jModeltest software with a bootstrap of 1000 replicates. Accession number, species, isolate, country and year are indicated for each strain. Different hosts are highlighted in different colors.

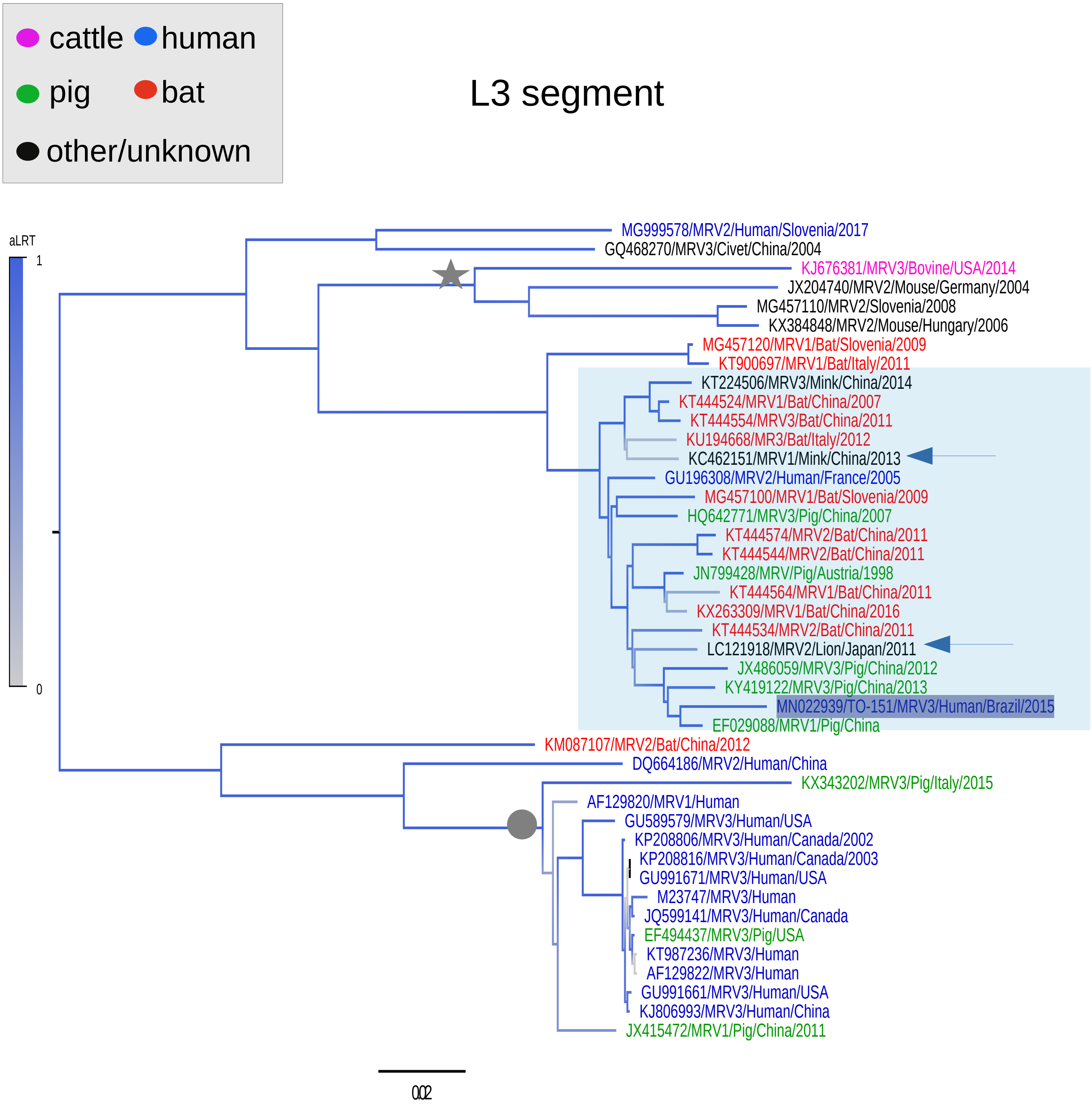

Figure S3. Phylogenetic tree inferred using the L3 segment of MRV3. Phylogenetic tree of the complete L3 segment (3184 bp) of the Brazilian TO-151/BR MRV3 strain (highlighted in the tree). The tree was performed using the maximum likelihood method GTR-G model within the jModeltest software with a bootstrap of 1000 replicates. Accession number, species, isolate, country and year are indicated for each strain. Different hosts are highlighted in different colors.

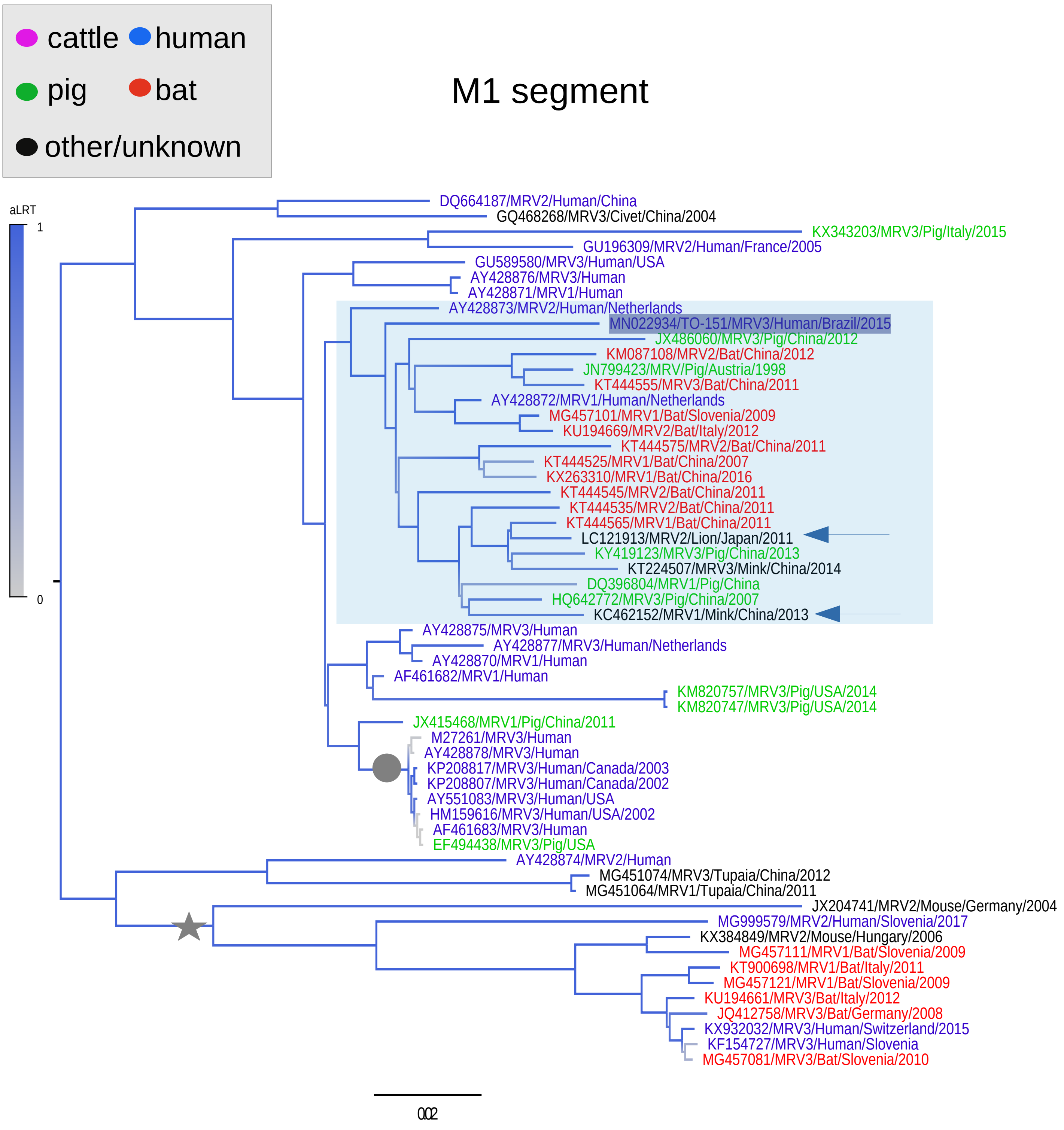

Figure S4. Phylogenetic tree inferred using the M1 segment of MRV3. Phylogenetic tree of the complete M1 segment (2253 bp) of the Brazilian TO-151/BR MRV3 strain (highlighted in the tree). The tree was performed using the maximum likelihood method GTR-G model within the jModeltest software with a bootstrap of 1000 replicates. Accession number, species, isolate, country and year are indicated for each strain. Different hosts are highlighted in different colors.

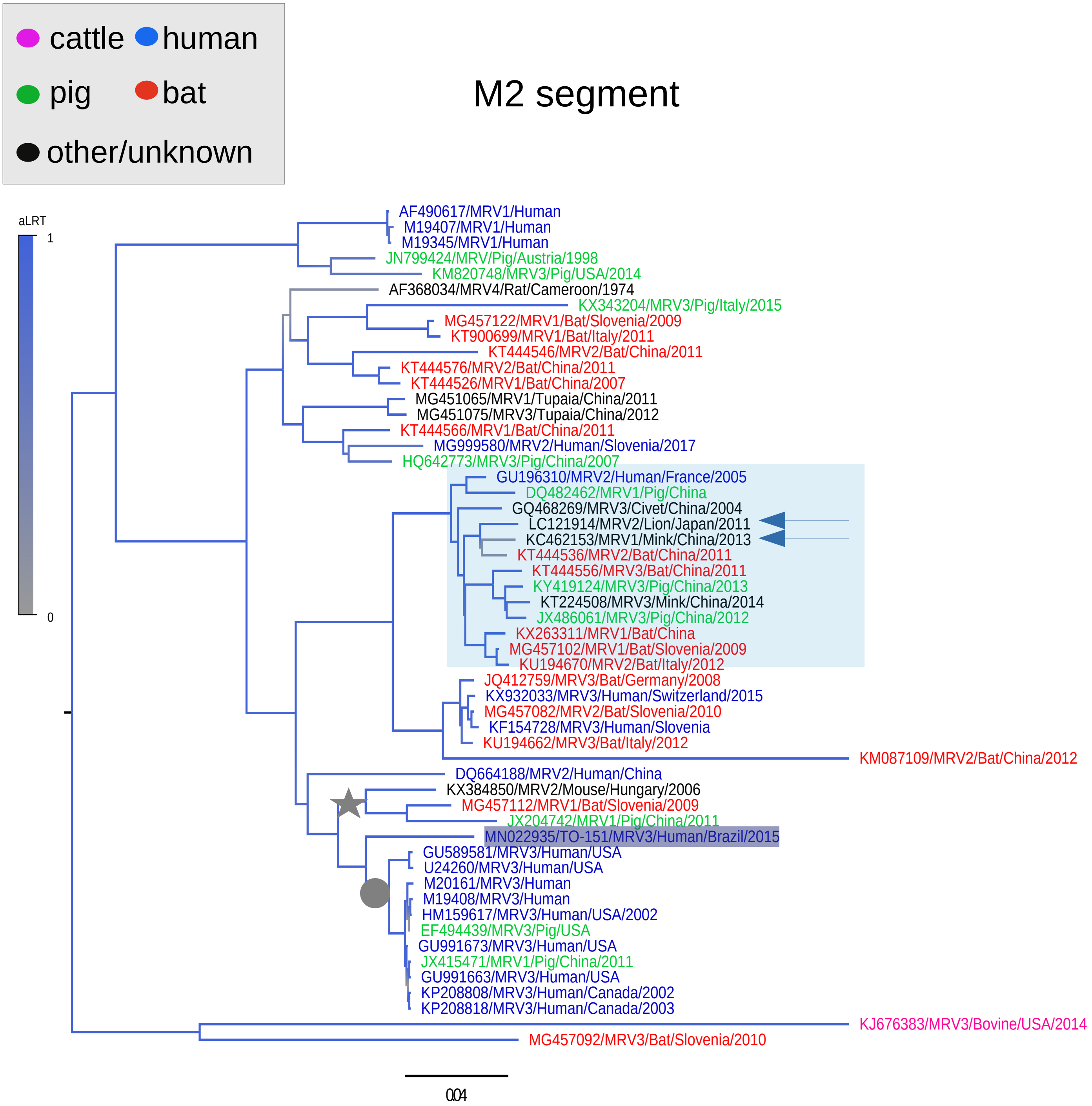

Figure S5. Phylogenetic tree inferred using the M2 segment of MRV3. Phylogenetic tree of the complete M2 segment (2154 bp) of the Brazilian TO-151/BR MRV3 strain (highlighted in the tree). The tree was performed using the maximum likelihood method GTR-G model within the jModeltest software with a bootstrap of 1000 replicates. Accession number, species, isolate, country and year are indicated for each strain. Different hosts are highlighted in different colors.

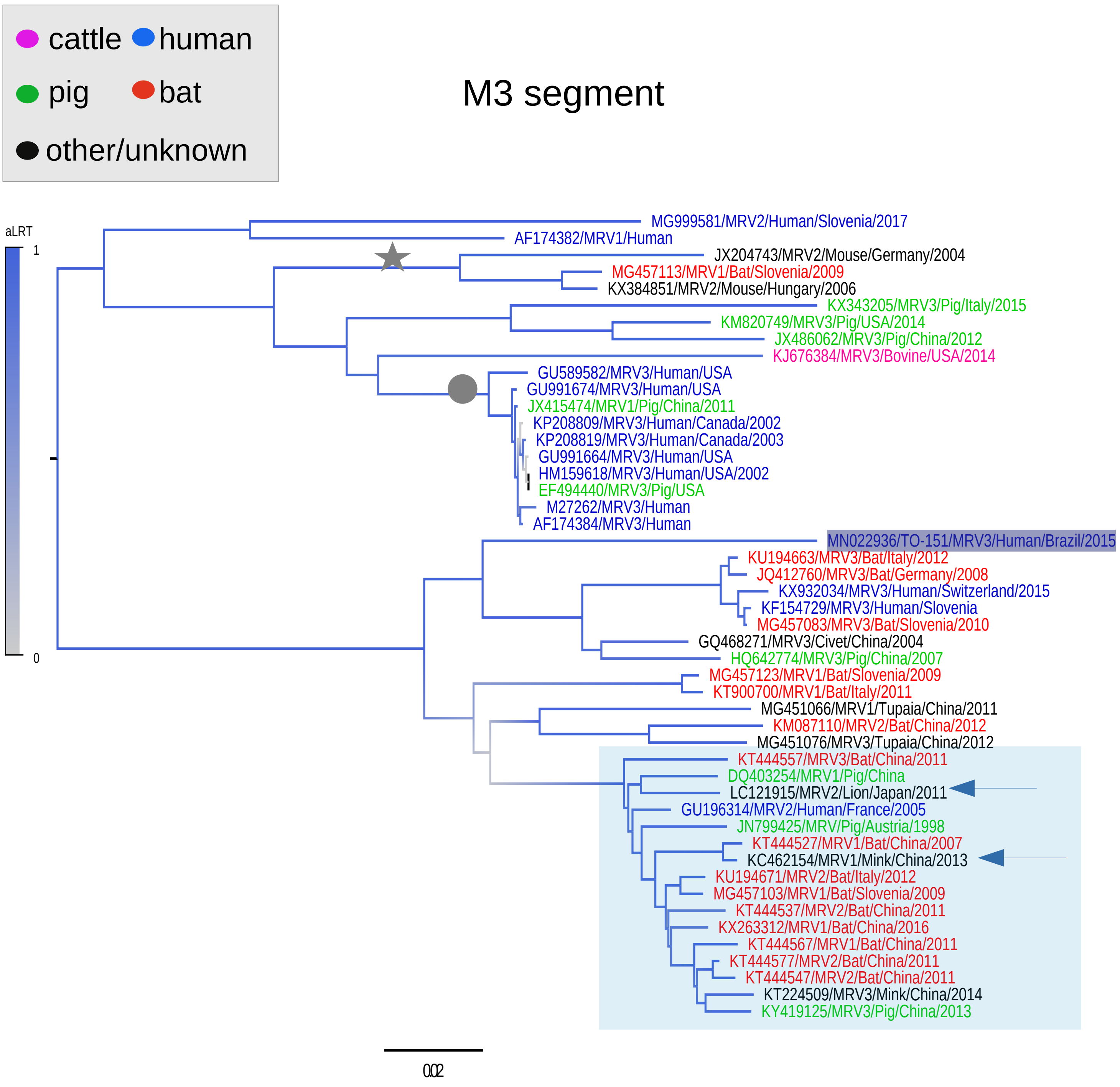

Figure S6. Phylogenetic tree inferred using the M3 segment of MRV3. Phylogenetic tree of the complete M3 segment (2336 bp) of the Brazilian TO-151/BR MRV3 strain (highlighted in the tree). The tree was performed using the maximum likelihood method GTR-G model within the jModeltest software with a bootstrap of 1000 replicates. Accession number, species, isolate, country and year are indicated for each strain. Different hosts are highlighted in different colors.

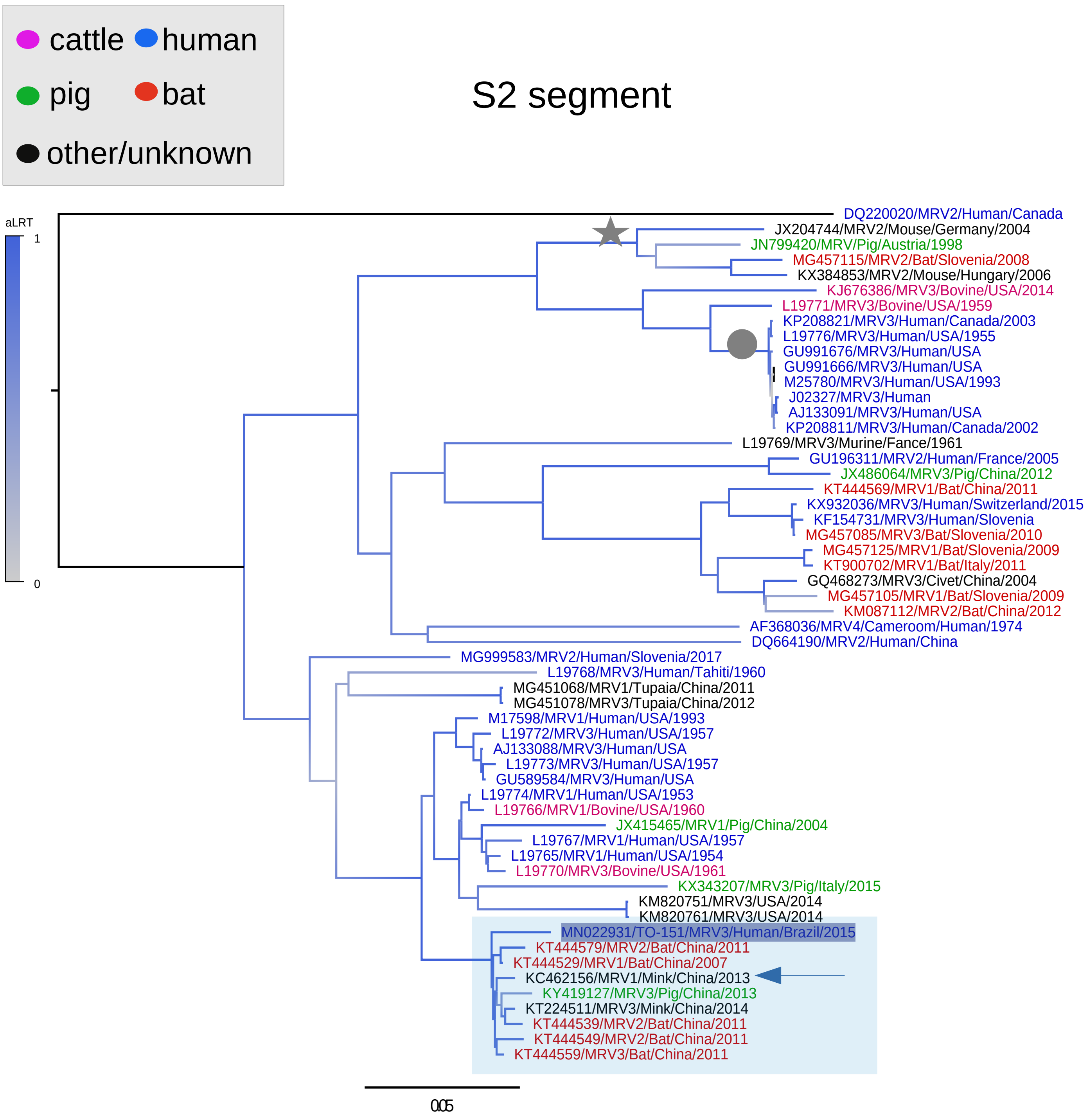

Figure S7. Phylogenetic tree inferred using the S2 segment of MRV3. Phylogenetic tree of the complete S2 segment (1320 bp) of the Brazilian TO-151/BR MRV3 strain (highlighted in the tree). The tree was performed using the maximum likelihood method GTR-G model within the jModeltest software with a bootstrap of 1000 replicates. Accession number, species, isolate, country and year are indicated for each strain. Different hosts are highlighted in different colors.

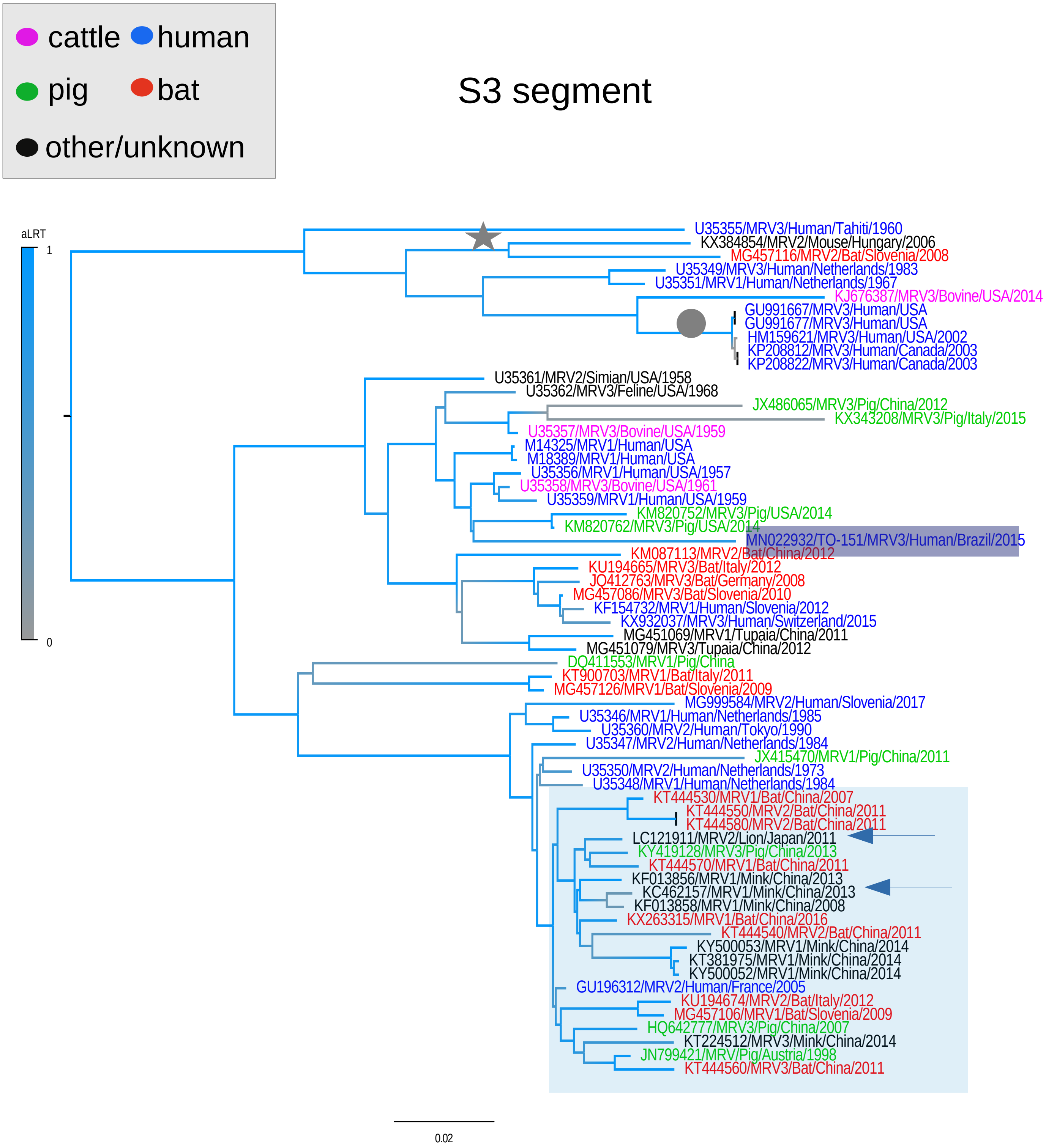

Figure S8. Phylogenetic tree inferred using the S3 segment of MRV3. Phylogenetic tree of the complete S3 segment (925 bp) of the Brazilian TO-151/BR MRV3 strain (highlighted in the tree). The tree was performed using the maximum likelihood method GTR-G model within the jModeltest software with a bootstrap of 1000 replicates. Accession number, species, isolate, country and year are indicated for each strain. Different hosts are highlighted in different colors.

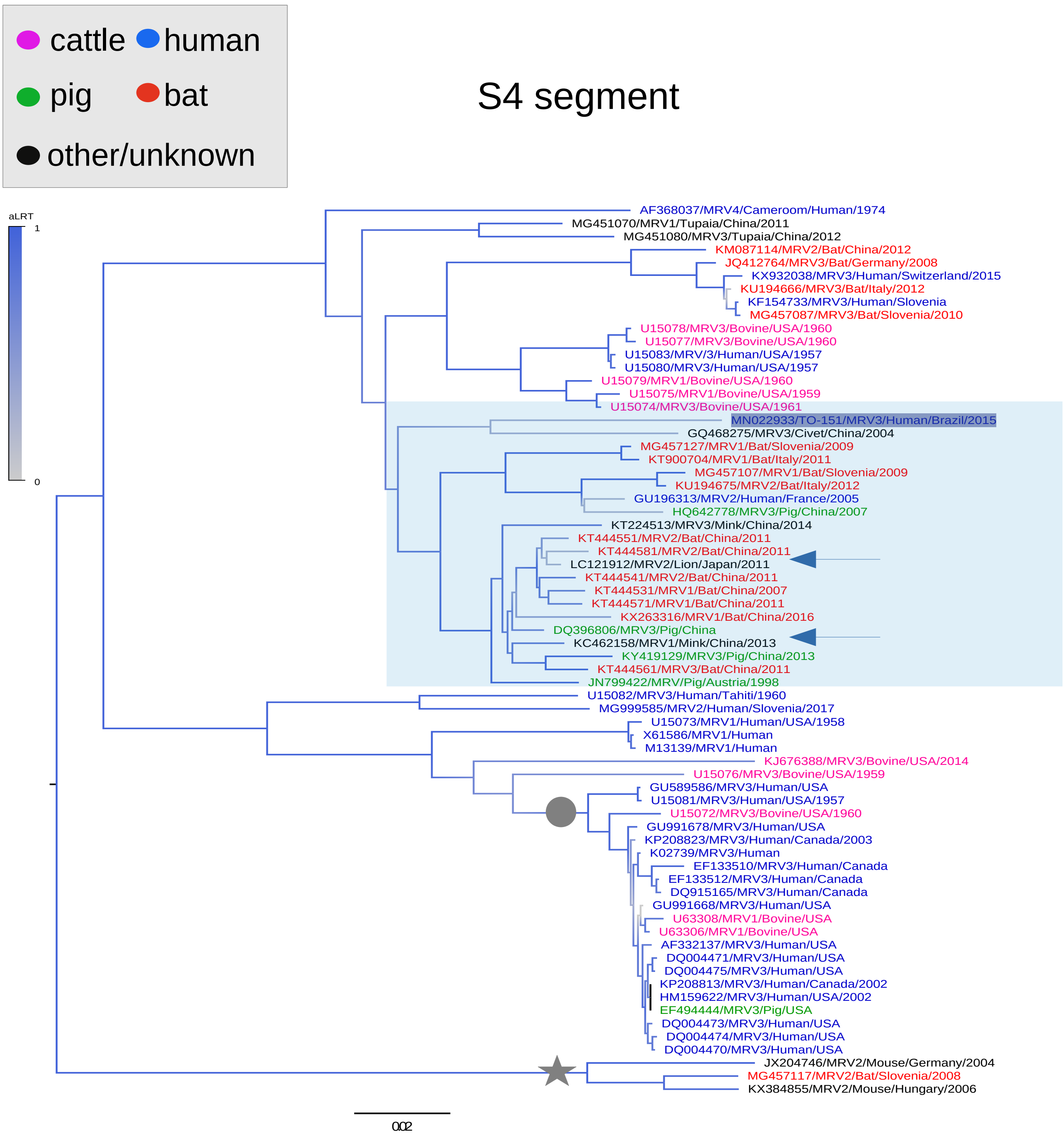

Figure S9. Phylogenetic tree inferred using the S4 segment of MRV3. Phylogenetic tree of the complete S4 segment (1104 bp) of the Brazilian TO-151/BR MRV3 strain (highlighted in the tree). The tree was performed using the maximum likelihood method GTR-G model within the jModeltest software with a bootstrap of 1000 replicates. Accession number, species, isolate, country and year are indicated for each strain. Different hosts are highlighted in different colors.

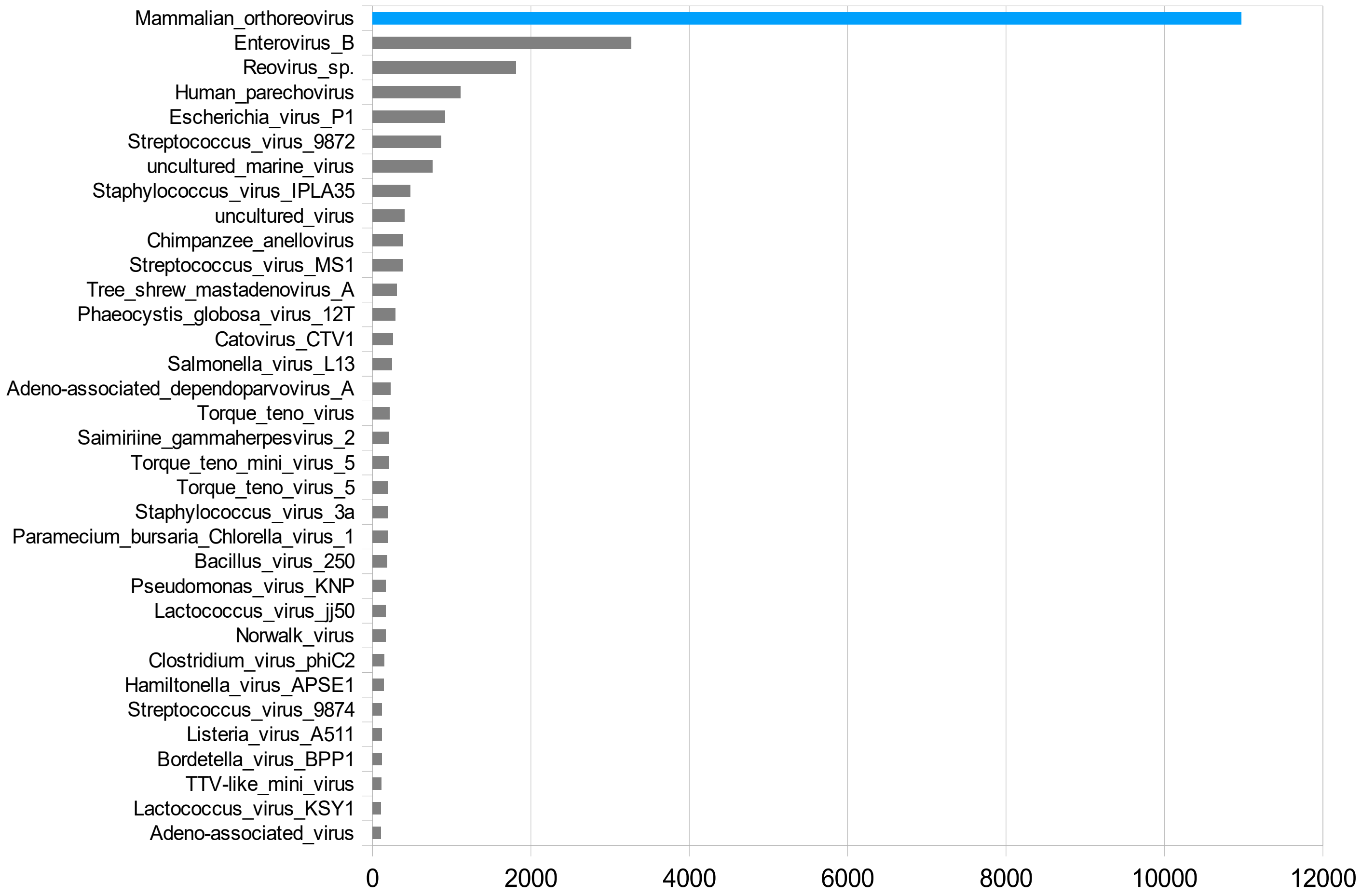

Figure S10. Virome of patient TO-151/BR. In the y-axis horizontal bars indicate all viruses detected in the patient TO-151/BR. In the x-axis the number of contigs of each virus found in the library is shown. The blue bar indicate the number of contigs of the MRV-3 detected in this library. Data were generated by the next generation sequencing of fecal sample (see the main text for details).

Accession number/Isolate Name/Host/Country/Isolation Year:

AY302467/T3/Human/Colorado/1996
AF368035/Ndelle/Murine/Cameroon/1974
JQ412761/T3/Bat/Germany/2008
KX343206/Pig/Italy/2015
JQ979272/Bat/Italy/2012
KX932035/Human/Switzerland
MG457084/Bat/Slovenia
KF154730/Human/Slovenia
JQ979276/Bat/Italy/2011
JQ979275/Bat/Italy/2011
L37676/France
JQ979271/T3/Bat/Italy/2011
JQ979273/Bat/Italy/2012
JQ979277/Bat/Italy/2011
JQ979283/Bat/Italy/2011
L37683/Human
L37684/Human
KKM820760/Pig/USA/2014
KM820750/Pig/USA/2014
L37679/MRV-3/Tahiti
AY860061/Canada
U53413/Cattle
X01161/MRV3
M10262/MRV3
JQ599138/Canada
EF133509/Canada
EF133511/Human/Canada
U53410/Cattle
U53409/Cattle
EF494441/MRV3
U53412/Cattle
U53411/Cattle
KP208810/Canada
GU991675/Human
KJ806994/Human
GU991665/MRV3
L37678/USA
U74291/Cattle
L37675/USA
GU589583/Human
L37680/Human
L37682/Human
U74293/Cattle
L37681/Human
U74292/Cattle
KY419126/Pig/China/2013
MN022930/TO-151/Human/Brazil/2015
DQ911244/Pig/China
JF829221/Pig/SouthKorea/2005
JF829220/Pig/SouthKorea/2005
JF829213/Pig/SouthKorea/2005
JF829222/Pig/SouthKorea/2005
JF829214/Pig/SouthKorea/2005
JF829219/Pig/SouthKorea/2005
JF829215/Pig/SouthKorea/2005
JF829218/Pig/SouthKorea/2005
JF829216/Pig/SouthKorea/2005
JF829217/Pig/SouthKorea/2005
KT224510/Mink/China/2014
JX486063/Pig/China/2003
KT444558/Bat/China/2011
GQ468272/Civet/China/2004
HQ642775/Pig/China/2007
MG451077/MRV-3/Treeshrew/China/2012
KP185124/Tupaia/China/2013
